# Surface Acoustic Wave Sensors for Hydrogen and Deuterium Detection

**DOI:** 10.3390/s17061417

**Published:** 2017-06-16

**Authors:** Aurelian Marcu, Cristian Viespe

**Affiliations:** National Institute for Laser, Plasma and Radiation Physics, Magurele 077125, Romania; aurelian.marcu@inflpr.ro

**Keywords:** hydrogen, nanowires, PLD-VLS, surface acoustic wave (SAW) sensors, gas sensor, surface acoustic wave

## Abstract

A delay-line-type surface acoustic wave (SAW) sensor based on a zinc oxide (ZnO) sensitive layer was developed. Two types of sensitive layers were obtained: ZnO nanowires and ZnO thin films, both deposited using laser methods (VLS-PLD and PLD, respectively) onto quartz substrates. The responses of sensors with two different nanowire lengths (300 and 600 nm) were compared with those of sensors with thin films of different thicknesses (ca. 100 and 200 nm) to different concentrations of hydrogen and deuterium. The experimental results revealed a high response at low concentrations and a rapid saturated response for nanowires, but a low response at low concentrations and a linear response to much higher gas concentrations for the thin-film-based SAW sensors.

## 1. Introduction

A high sensor sensitivity and short response time are key in gas detection. Increasing the sensitive surface area of the surface acoustic wave (SAW) sensors is one of the most common approaches to address these problems. Nanostructured [1,2,3,4,5] active layers are frequently used to improve sensor performance. A sensor response usually depends on the quantity of the gas retained by the active layer, and such layers offer much greater adsorption of a targeted gas than conventional thin films. SAW sensors have been used with optimal results for the detection of different gases, such as hydrogen [6,7,8], CO_2_ [9], and CH_4_ [10], detection of humidity [11,12], etc. Deuterium was detected with a SAW sensor here for the first time. It has previously been reported that SAW sensors can well detect hydrogen using sensitive films of different materials such as ZnO_3_ [13,14,15], Pd [16,17], and WO_3_-Pd [18].

Previous work [13,19] has demonstrated that sensor response is significantly enhanced by nanostructured surfaces, in particular by vertically aligned nanowires grown on a sensor’s active surface area. These designs allow detection of up to a 10-fold lower gas concentration (in particular for hydrogen detection [15]) compared with the thin film designs. The considerably larger active surface area provides a significantly greater response to lower gas concentrations, and one might be tempted to conclude that superior sensor performance would result from increasing the active surface area. However, if the nanowires are too long, the sensor does not oscillate at all, and the sensor is thus non-functional [15]. Furthermore, the frequency shift variation in nanowire-based sensors, unlike thin-film sensors of greater thickness [15], does not exhibit a linear response, even at comparably active surface volumes and measuring conditions. There are clearly fabrication and utilization limitations that need to be considered for the development of sensors with larger active surfaces that are suitable for measuring a particular gas under specific conditions. The gas retention mechanism of the active layer is considered to be essential in addressing these limitations.

The aim of the present work was to experimentally compare the response of superficial adsorption-based SAW sensors with volume absorption-based SAW sensors and to note the specific advantages and drawbacks of each type.

## 2. Materials and Methods

**Figure 1:**
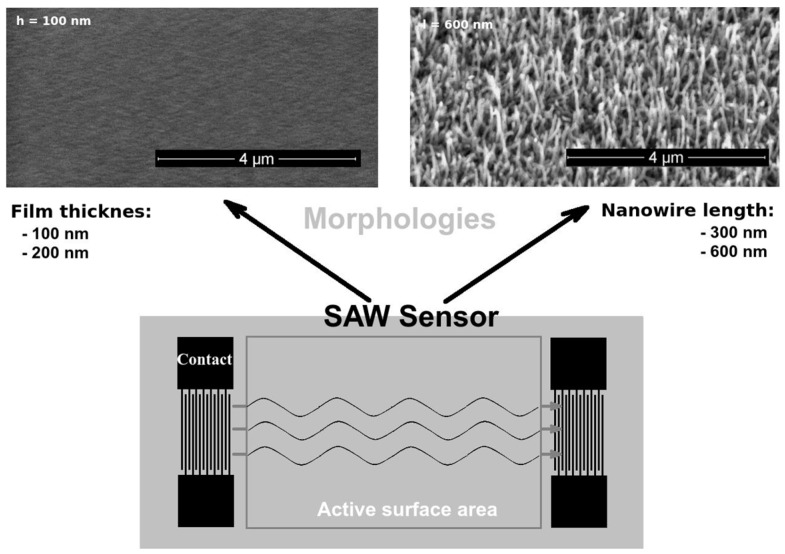
SAW sensor design and surface morphologies of the active area.

The SAW sensor was of a delay-line type and based on a ST-X-cut quartz substrate, with an oscillating frequency of 69.5 MHz (Figure 1). The interdigital transducers (IDT) were made by photolithographic techniques from a 150-nm-thick gold layer, with a 10-nm-thick chromium layer to assure adhesion to the quartz substrate. Each IDT pattern consisted of 50 pairs of fingers, with a periodicity of 45.2 μm and a 2500 μm wide acoustic aperture. The active area was 8 × 10 mm^2^, and the quartz substrate of 10 × 38 mm^2^ was cut in a parallelogram configuration with a 45° angle to reduce the reflection of acoustic waves on the edge of the quartz substrate.

The pulsed laser deposition (PLD) technique was used to deposit 100- and 200-nm-thick crystalline ZnO thin films on the SAW sensor active surface area. Furthermore, 300- and 600-nm-long ZnO single-crystal nanowires (diameter: 20–40 nm) were grown on the active surface area using PLD in a plasma reflection plume filtering configuration [20] and vapor–liquid–solid (VLS) techniques, respectively [21] (Figure 1). Details regarding nanowire growth parameters and nanowire-based sensor fabrication techniques are reported elsewhere [15,22,23,24].

The two nanowire-based sensors had similar material compositions (ZnO) and structures (single-crystal nanowires) when our PLD/VLS method was used [25]. The gold catalyst was simultaneously deposited on both sensors and provided similar coverage (filling factor); a coverage percentage p_r_ of about 25% of the grown area was estimated. The two sensors had a similar nanowire diameter, ranging from 20 to 40 nm; the tapering of a few hundred nanometers in nanowire length was negligible under our experimental conditions. Thus, the only significant difference between the two sensors was the nanowire length. Due to the vast surface area compared with a thin film type of sensor, gas (in our case, hydrogen isotopes) retention was mainly by “superficial adsorption” since the surface/volume ratio of the nanowires is very large. This is because, observing from the active surface area plane, the depth of the layer did not exceed 10–20 nm (the maximum distance from each in-depth point to the closest interface plane), which was about 10–20 times smaller than the thin film thicknesses of the fabricated film-based SAW sensors. For the nanowire sensors, the ‘additional’ sensitive area (compared with that of the film sensors) was the lateral area of the nanowires, which could be roughly approximated as:

S_nm_ = 2 N π r h (1)

where S_nm_ is the nanowire active (lateral) surface area, N is the number of wires, r is their radius, and h is their length. Furthermore, the number of nanowires in the sensor active area was estimated as follows:

N = (p_r_ L W)/(π r^2^) (2)

where p_r_ is the percentage of the nanowire area covered (ca. 25%), and L and W are the length and the width, respectively, of the active area (in our case, about 8 × 10 mm^2^). Using Equations (2) and (3), the nanowire active area surface is approximated by

S_nm_ = (2 h p_r_ L W)/r. (3)

In our case, we can assume an average diameter of 30 nm and estimate about 8 × 10^−4^ m^2^ as the ‘extra’ area added by the 300-nm-long nanowires to the nanowire ‘global’ area. This active area was approximately 10 times larger than that of the thin-film sensor, which was estimated at 8 × 10^−5^ m^2^. Consequently, for 600-nm-long wires, the added surface was about 20 times larger (ca. 1.6 × 10^−3^) than that of a thin film.

The thin film active layer volumes are given by the following:

V_f_ = L W h_f_ (4)

and were 1.2 × 10^−11^ and 2.4 × 10^−11^ m^3^ in our case. For our nanowire sensors, Equation (5) given below was used to estimate mean volumes of 6 × 10^−12^ and 1.2 × 10^−11^ m^3^.

V_n_ = N π r^2^ h_n_. (5)

Consequently, the 100-nm-thick thin film had a volume that was comparable to that of the 600-nm-long nanowire covered area, while the surfaces were in a 1:20 ratio.

The responses of the SAW sensors to hydrogen and deuterium gases were measured at room temperature (~24 °C). Their retention processes were expected to be similar because different isotopes of an atom will generally display similar behaviors in their physical and chemical interactions. The sensor frequency shift is known to be proportional with the absorbed gas mass [26], according to Equation (6):

Δf = −k S_m_ Δm (6)

where f is the resonant frequency, S_m_ is a device specific constant, and k is the fraction of the area affected, which in our case was unity for the total area surface. However, the atomic mass of deuterium is double that of hydrogen. Thus, the two gases were expected to generate a double frequency shift for the two isotopes under comparable test conditions.

**Figure 2:**
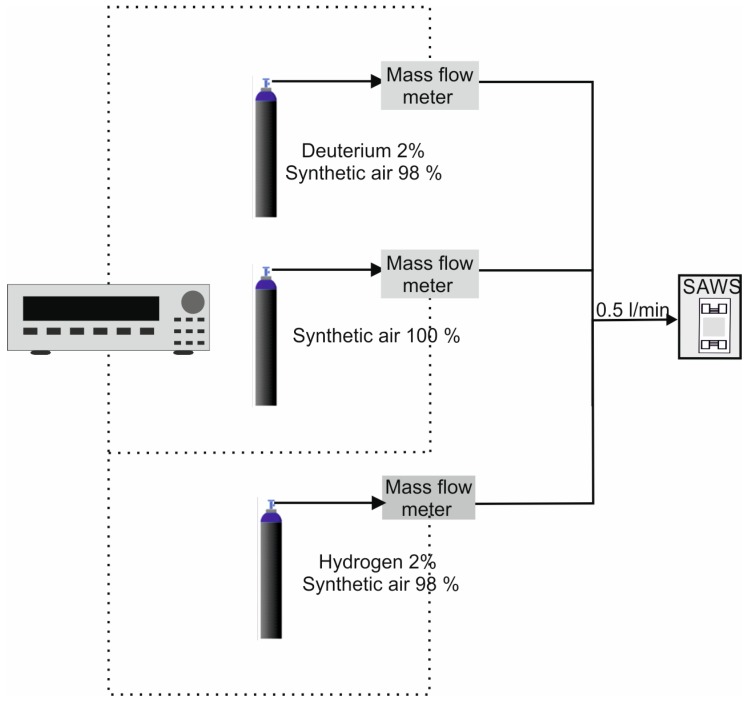
Experimental setup.

Figure 2 shows the gas concentration measurement setup, consisting of three gas cylinders: two containing deuterium and hydrogen at a concentration of 2% in synthetic air and one containing synthetic air. The gas concentration was controlled using an MKS controller connected to three mass-flow meters, one for each cylinder. The flow rate was maintained constant at 0.5 L/min, regardless of the hydrogen (isotope) concentration.

The oscillating system of the SAW sensor included an amplifier (Model DHPVA-100, FEMTO Messtechnik GmbH; 10–60 dB, 100 MHz, Berlin, Germany), a band-pass filter (Model B9336, Anatech Electronics Inc., Garfield, NJ, USA), and a phase shifter (Model IF-70-360-S, I.F. Engineering Corp., Dudley, MA, USA). The frequency shift of the system was read with a frequency counter/analyzer (Pendulum CNT-91, Spectracom Corp., Rochester, NY, USA) using TimeView 3 software (Spectracom Corp., Rochester, NY, USA).

## 3. Results

**Figure 3:**
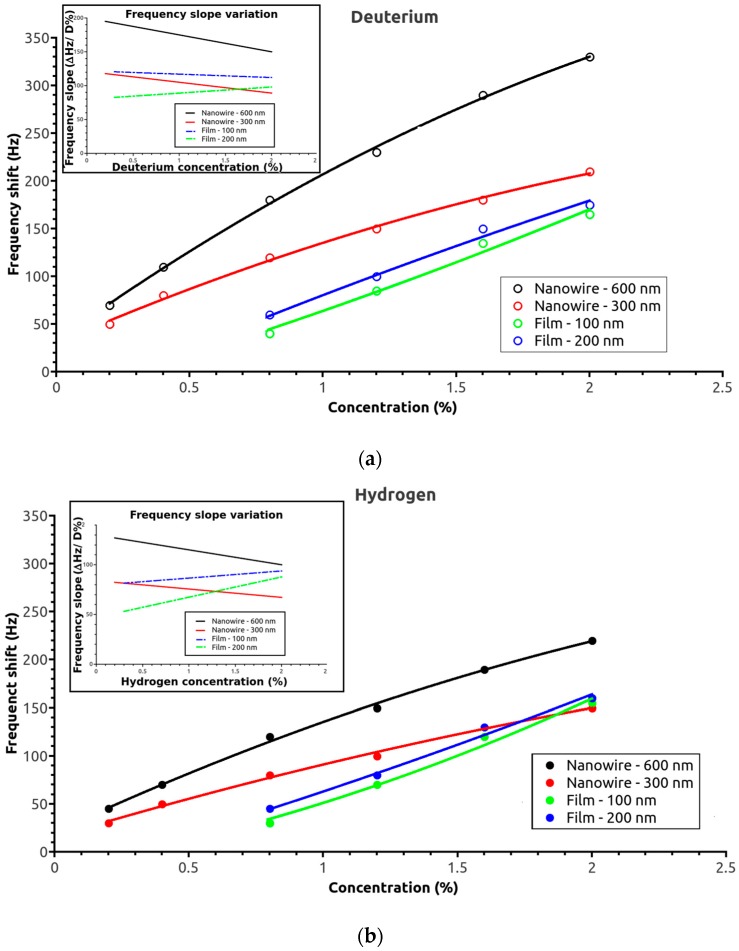
Frequency shifts of the sensors as a function of (**a**) deuterium concentration (inset: variation in frequency shift slope as a function of deuterium concentration) and (**b**) hydrogen concentration (inset: variation in frequency shift slope as a function of hydrogen concentration).

The SAW sensor response was measured for the four SAW sensor active surface morphologies for hydrogen and deuterium concentrations up to 2% (half of the safety limit for this gas). The frequency shift dependences as a function of deuterium concentration for the four sensors are shown in Figure 3a. As expected, the nanowire-based sensors had a higher response at low deuterium concentrations, while detection for the thin-film sensors started at ca. 0.3%. The frequency shift increased continuously with deuterium concentration for all of the sensors. The 600 nm sensor gave the strongest signal over the entire measured range. For the thin films, the 200 nm film sensor also had a higher response than the 100 nm one; however, the difference was not as great as that of the two nanowire-based sensors. Notably, the frequency shift slopes for the two sensor morphologies were quite different: The slope continuously decreased over the measured interval with the nanowire sensor, while it remained nearly constant or slightly increased with the thin film one (Figure 3a inset). The trends were the same with hydrogen gas: a higher response was measured for the nanowire sensors at lower hydrogen concentrations. Steadily increasing values were observed with the thin-film sensors, whereas, with the nanowire sensors, the slopes steadily decreased as hydrogen concentration increased. However, the overall responses of the sensors were lower for hydrogen, and the responses of the thin-film sensors were slightly higher than that of the 300 nm nanowire sensor at concentrations near 2% (Figure 3b).

Table 1 lists the sensitivities and detection limits for the different sensitive layers. The limit of detection (LOD) depends on the noise level, being defined as 3 × noise level/sensitivity. The noise level was estimated at ca. 10 Hz for the nanowires and 8 Hz for the films. The noise assessment was performed in air (without analyte) by measuring the frequency fluctuation over 10 min; this represents the maximum frequency deviation from the trend line (best fit line). The sensitivity, defined as the frequency shift in Hz per unit analyte concentration in ppm, was determined from an average sensitivity value for a gas concentration between 0.3 and 2%. The global sensitivity of the nanowire-based sensors was higher than that of the film-based sensors. The LODs obtained for the 600 nm nanowire sensor were 2117 ppm for hydrogen and 366 ppm for deuterium. To determinate the responses and recovery time, the frequency change of the SAW sensor was measured at different gas concentrations between 0.3 and 2% with the respect of the baseline using synthetic air as a reference. The response time (necessary to reach 90% of maximal signal) for the nanowire sensors was between 9 and 15 s, and for the films sensor was 2–3 s slower. The recovery time, for the nanowire sensors, was between 6 and 9 s and, for the film sensors, was between 7 and 11 s.

## 4. Discussion

**Figure 4:**
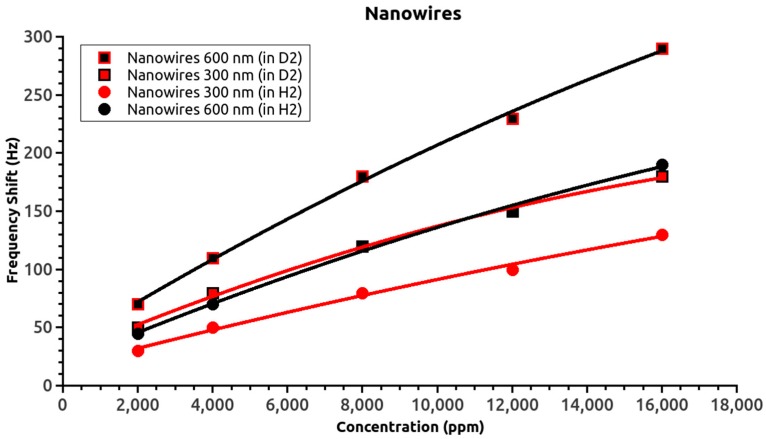
Nanowire sensor response as a function of hydrogen isotope concentration.

Figure 4 shows the frequency shifts of the nanowire-based SAW sensors for both isotopes. The shorter nanowire sensor response for deuterium overlapped with the longer nanowire sensor response for hydrogen. Thus, considering the proportionality of the frequency shift with the absorbed gas mass, the 1:2 surface ratio of the active layers, and the 2:1 ratio of the gas atomic mass, the overlap of the two curves confirmed the reliability of the obtained data. The thin-film sensor data also supported this conclusion; however, the overlap of the thin film curves is less relevant due to the smaller frequency shift with changing hydrogen isotope.

Comparison of the nanowire and thin film responses for the same gas concentration revealed differences in frequency shift slopes. The slopes for the film sensors could be fitted by a linear equation (see the inset), but the nanowire sensors slopes could not. The difference in sensor behavior was attributed to the increase in the absorbed gas weight and/or differences in the hydrogen retention processes. Previous experiments [15] showed that increasing the nanowire length or diameter (and therefore weight) rapidly led to complete absorption of gas molecules by the sensitive layer of the SAW devices, which caused the sensor to stop oscillating, i.e., stop functioning. In the present case, the frequency shift tended to saturate with increasing hydrogen (isotope) concentration. This suggested that the differences must be related to the hydrogen retention process rather than the weight-induced changes of the nanowires oscillations.

There are two main physical explanations for gas retention by the sensor active layer: superficial adsorption and volume absorption. For superficial adsorption, Langmuir’s law states that the variation in occupancy sites is an asymptotic function of the gas partial pressure variation and can be modeled by a function of the form:

θA = P/(P + P_H_) (7)

where θA is the surface sites occupancy, P is the ambient pressure, and P_H_ is the hydrogen (isotope) partial pressure. The conceptual basis for this adsorption model is a continuous monolayer of adsorbate molecules covering a homogeneous solid surface [27]. Such a model essentially explains the saturated variation in the gas retention process with the hydrogen (isotope) partial pressure and provides a sensor response fitting function of the following form:

Δf = A (1 − B/(B + P_H_)) (8)

where A and B are constants corresponding to our experimental case. The experimental points from all of our nanowire SAW sensors were reasonably fitted by such a function.

For the volume absorption process, Fick’s first law describes diffusion as

J = −D d φ/dx(9)

where J is the diffusion flux, φ is the gas concentration, x is the spatial coordinate (in our case depth from the surface), and D is the diffusion coefficient. Fick’s law predicts that a gas will gradually diffuse (with decreasing speed) until the gas concentration equilibrates to the ambient concentration in the absorbing volume. However, when diffusion occurs into a solid material, the diffusion coefficient is not a simple value but a specific material coefficient that follows the Arrhenius equation of the following form:

D = D_0_ e^−Ea/(kT)^ (10)

where D is the (effective) diffusion coefficient (as in Equation (9)), D_0_ is the maximal diffusion coefficient (at infinite temperature) (m^2^/s), T is the absolute temperature, k is Boltzmann’s constant, and Ea is the activation energy (J/atom). Considering the variation in the diffusion coefficient and the presence of a threshold activation energy, diffusion into a solid material should gradually decrease with depth into the solid material volume. However, experiments performed on the crystalline ZnO have shown that both (electrochemical) injection and diffusion of hydrogen significantly affects the retained volume hydrogen concentrations (between 2% and 5%) for thicknesses up to about 300 nm [28]. Thus, we approximated it as a constant in our active thin layer volume and, as such, dependent only on the gas partial pressure. In this case, Equation (9) simplifies to an absorption process that is directly proportional to the hydrogen (isotope) partial pressure, and the absorbed gas into the volume will vary quasi-linearly. This dependence should be true at least at low hydrogen partial pressures, which was in agreement with our experimental values up to 2%.

Summarizing the above physical models, surface adsorption would provide a saturated variation in the retained gas (and mass) while volume retention would behave quasi-linearly. For example, consider the responses of the 600-nm-long nanowire sensor and the 100-nm-thick thin film sensor: these had a comparable volume of active ZnO material, but a 1:20 ratio of the layer surface. The retained hydrogen (isotope) mass in volume would be comparable, but the surface-retained one would be at the 20:1 ratio. Thus, if, for a low hydrogen concentration, the absorption into volume is rather insignificant while surface adsorption is rather dominant, the nanowire SAW sensor response will dramatically improve. As the partial pressure and the volume absorption begins to increase, the sensor response will gradually become comparable to the surface retained case. In this way, the nanowire sensor response will change slightly with increasing hydrogen partial pressure. With respect to the thin films 200 nm or thicker [15], the increase in gas absorption will be relatively significant and might actually slowly overcome the hydrogen (isotopes) surface retained mass.

**Figure 5:**
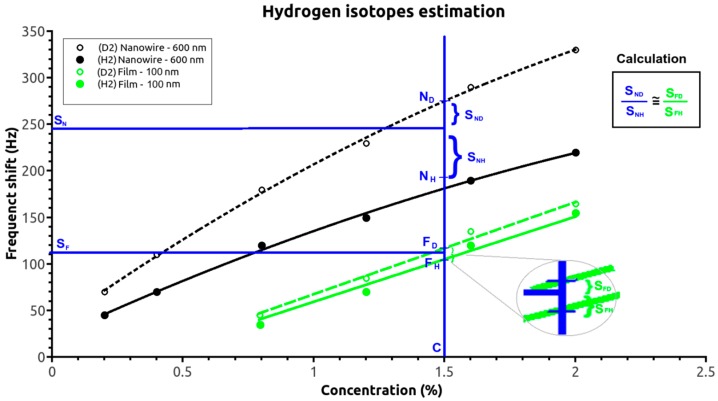
Sample calculation to estimate the hydrogen isotope concentration.

The non-linear frequency shift response of the surface adsorption-based sensors suggests that using two sensors could distinguish other gases; the current sensors distinguished two hydrogen isotopes. Figure 5 shows the 600-nm-nanowire-based and 100-nm-film-based SAW sensor responses for different hydrogen and deuterium concentrations and the specific responses (S_F_ and S_N_) for a given hydrogen/deuterium mixture. Using a quasi-linear response of the SAW sensor with the retained gas mass (Equation (6)), the two frequency shifts must be placed between the two response curves (corresponding to only deuterium and only hydrogen) of the two SAW sensors. Furthermore, the position of the two points can be approximated as being at proportional distances between the two extremes:

(N_D_ − S_H_)/(S_H_ − N_H_) = (F_D_ − S_F_)/(S_F_ − F_H_) (11)

where N_D_, N_H_, F_D_, and F_H_ are the frequency shifts of the nanowire- and film-based SAW sensors for deuterium and hydrogen, respectively, and S_N_ and S_F_ are the nanowire and film sensor responses for a given hydrogen isotope mixture.

Thus, if the N_D_ and N_H_ points are assigned to linear (D and H) calibration curves (for the thin-film sensor according to Equation (9)), F_D_ and F_H_ points are assigned to the saturated variations curves (for the nanowire sensor according to Equation (8)), and a proportional relationship is assumed between the measured S_F_ and S_N_ frequency shifts and the reference curves (N_X_ and F_X_) points for the two isotopes (according to Equation (11)). The hydrogen and deuterium concentrations in the ambient gas can then be readily calculated (both analytically and numerically).

## 5. Conclusions

In conclusion, 300- and 600-nm-long nanowires and 100- and 200-nm-thick thin film SAW sensor responses were measured for various hydrogen and deuterium gas concentrations. The surface adsorption-based and volume absorption-based SAW sensor responses were compared for an equivalent sensor active layer volume and a 1:20 ratio of the active surface layers, i.e., for the 100-nm-thick thin film and the 600-nm-long nanowire. The experimental results revealed a high response at low concentrations and a rapid saturated response for the surface adsorption-based sensors, but a low response at low concentrations and a linear response to much higher gas concentrations for the volume absorption-based SAW sensors. The reliability of the experimental results was confirmed by the overlapping response curves of two sensors with a 1:2 ratio of the active layer volumes (and areas) and a 2:1 ratio of gas atomic masses. Our study of the hydrogen isotopes suggests that the nonlinearity of the response curves when different gases are retained by a surface adsorption-based SAW sensor’s active layer could be exploited for gas identification.

## Figures and Tables

**Table 1:** Sensitivity and LOD (Δf = frequency change; f = resonance frequency; c = concentration).

	Hydrogen		Deuterium	
Sensitive layers	Sensitivity Δf/c [Hz/ppm]	LOD [ppm]	Sensitivity Δf/c [Hz/ppm]	LOD [ppm]
Nanowire 300 nm	0.01	3115	0.059	539
Nanowire 600 nm	0.015	2117	0.09	366
Film 100 nm	0.005	2303	0.026	747
Film 200 nm	0.004	2802	0.023	1004
